# Eradication of *Aspiculuris tetraptera* in various immunodeficient mouse models using ivermectin: a case report

**DOI:** 10.1186/s42826-025-00263-5

**Published:** 2026-01-23

**Authors:** Ji-Hun Lee, Eun-Seon Yoo, Na-Won Kim, Han-Bi Jeong, Ah-Reum Kang, Sun-Min Seo, Young-Jun Park, Byeong-Cheol Kang, Yang-Kyu Choi

**Affiliations:** 1 Department of Laboratory Animal Medicine, College of Veterinary Medicine, Konkuk University, Seoul, 05029, South Korea; 2 Department of Experimental Animal Research, Biomedical Research Institute, Seoul National University Hospital, Seoul, 03080 South Korea

**Keywords:** *Aspiculuris tetraptera*, Ivermectin, Pinworm, Facility management, Immunodeficient mouse

## Abstract

**Background:**

Despite advancements in laboratory animal facility management, pinworm infections remain a persistent issue in immunodeficient mouse colonies. Rapid diagnosis and treatment are crucial to mitigating potential scientific and economic consequences. Effective control requires both the administration of anthelmintic agents and rigorous environmental decontamination. However, the safety and efficacy of these treatments in genetically modified mouse models remains uncertain.

**Case presentation:**

*Aspiculuris* *tetraptera* infestation was identified in multiple immunodeficient mouse models housed in a laboratory facility. Diagnosis was confirmed through fecal flotation for egg detection and necropsy for adult worm examination in the large intestines. Mice received three subcutaneous ivermectin injections at two-week intervals, coupled with environmental decontamination using ivermectin spray for four consecutive weeks. Following treatment, all colonies tested negative for *A. tetraptera* without any mortality.

**Conclusions:**

A combination of subcutaneous ivermectin injection and environmental spray application effectively eradicated *A. tetraptera* infestation in immunodeficient mouse colonies. The treatment protocol led to the complete elimination of eggs and adult worms, offering a practical strategy for managing pinworm infections in genetically modified mouse models. Limitations include the small sample size, and the lack of a comprehensive evaluation of physiological and metabolic safety in immunodeficient mice. Further validation will be required to confirm the broader applicability of this approach.

## Background

Detecting pathogens in laboratory animals is crucial for maintaining their health, which directly impacts the reliability and quality of experimental outcomes [1, 2]. Consequently, routine health monitoring systems are widely implemented in animal research facilities to identify pathogen infections [3]. Despite advancements in facility management, pinworm infections remain prevalent in laboratory mouse colonies [4, 5]. Among these, oxyurid pinworms are the most common, with *Syphacia muris*,* Syphacia obvelata*, and *Aspiculuris tetraptera* being the primary species monitored in specific pathogen-free (SPF) programs [6]. While these parasites generally do not cause overt clinical symptoms in mice, they can influence physiological states and potentially interfere with experimental results [7].

To prevent both economic and scientific losses, a well-structured eradication program for pinworm infections is essential. Successful eradication strategies typically involve targeted administration of anthelmintic agents, considering the pinworm life cycle [8]. Furthermore, thorough decontamination of the facility environment using effective disinfectants is necessary to minimize the risk of reinfection.

With the growing demand for diverse animal models, genetically modified mice, including knockout models, have become indispensable in biological and medical research. However, inadequate facility management can expose these genetically modified mouse colonies to pathogens. Given the limited research on the consequences of such exposures, their potential impact on experimental outcomes remains uncertain [9, 10]. Therefore, rapid diagnosis and the implementation of an effective eradication program are crucial for preserving research integrity.

Here, we present a case of *A. tetraptera* infection in various immunodeficient mouse colonies housed in laboratory facilities and describe the successful eradication of the infection using ivermectin.

## Case presentation

### Mouse models

All affected mice had a C57BL/6 genetic background and ranged from 2 to 30 weeks old. They were originally obtained from SPF facilities, and no other pathogens listed in the SPF health monitoring program were detected. The various immunodeficient models housed in our facility are summarized in Table 1. All procedures were approved by the Institutional Animal Care and Use Committee (approval number: KU24204) and were conducted in accordance with the ARRIVE guidelines.

**Table 1:** Immunodeficient mouse colonies maintained in our facility

Immunodeficient Mouse Model	Number of Cages
Rag1^−/−^ mouse	6
Rag2^−/−^ mouse	9
C1qa^−/−^ mouse	18
Rag1^−/−^, C1qa^−/−^ mouse	3
Rag2^−/−^, C1qa^−/−^ mouse	10
TLR4^−/−^, TLR9^−/−^ mouse	11
TNF-α^−/−^ mouse	4
IL-10^−/−^ mouse	7
IL2RG^−/−^ mouse l	14

### Husbandry conditions

The mice were housed in a semi-SPF facility within an individually ventilated cage system (Three-Shine Inc., Korea), with 4–6 mice per cage. The facility environment was maintained at a temperature of 21–24 °C, humidity of 40%–60%, and a 12-hour light/dark cycle. The animals were provided with autoclaved Sani-Chips bedding (Koatech, Korea) and had access to sterile diets (Koatech, Korea) and water *ad libitum*. Facility technicians followed strict biosecurity measures, including the use of personal protective equipment (PPE) such as protective clothing, masks, latex gloves, and shoes sanitized with 70% alcohol. Equipment introduced into the facility was either autoclaved or disinfected to prevent contamination.

### Diagnosis

**Fig. 1:**
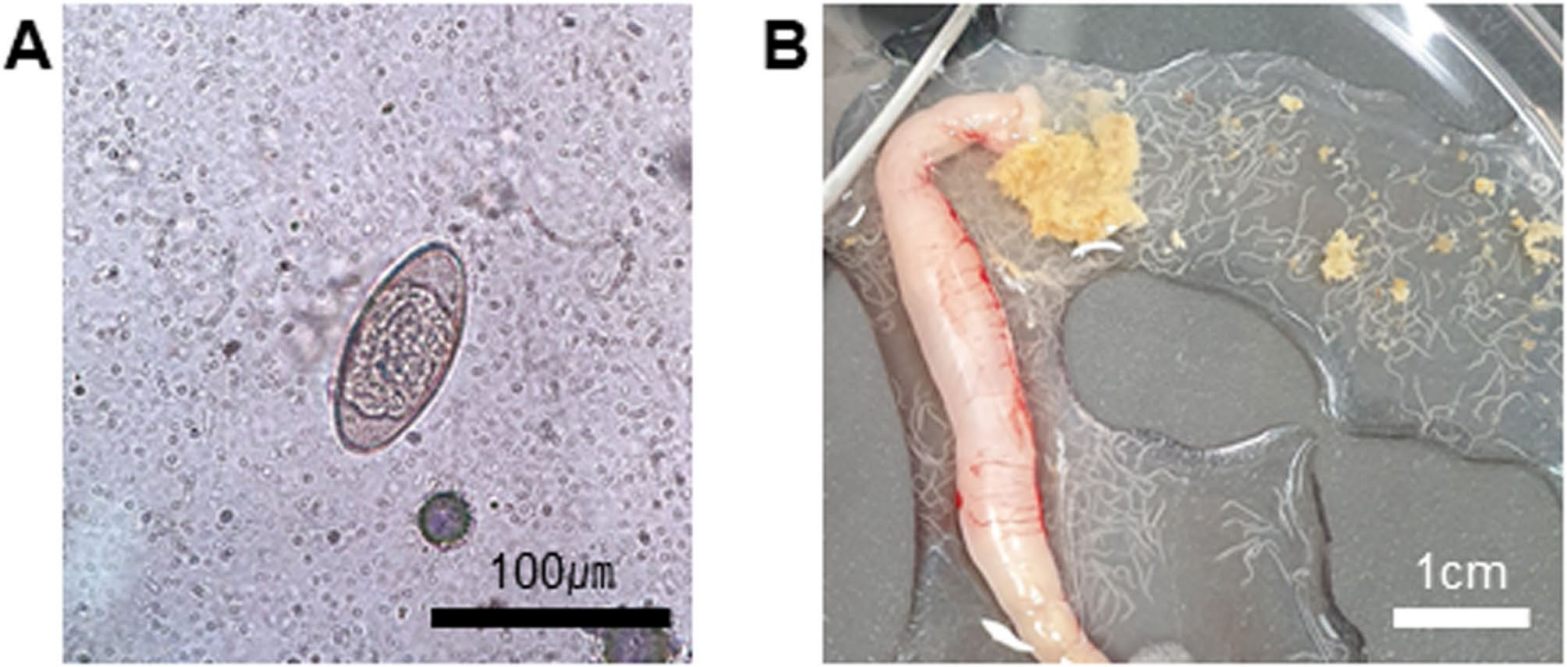
Diagnosis of *Aspiculuris tetraptera* infestation. (**A**) *A. tetraptera* egg examined under a light microscope at 400× magnification, displaying a bilaterally symmetrical shape. Scale bar = 100 μm. (**B**) Adult *A. tetraptera* worms found in the large intestine of an infected mouse. Scale bar = 1 cm

**Fig. 2:**
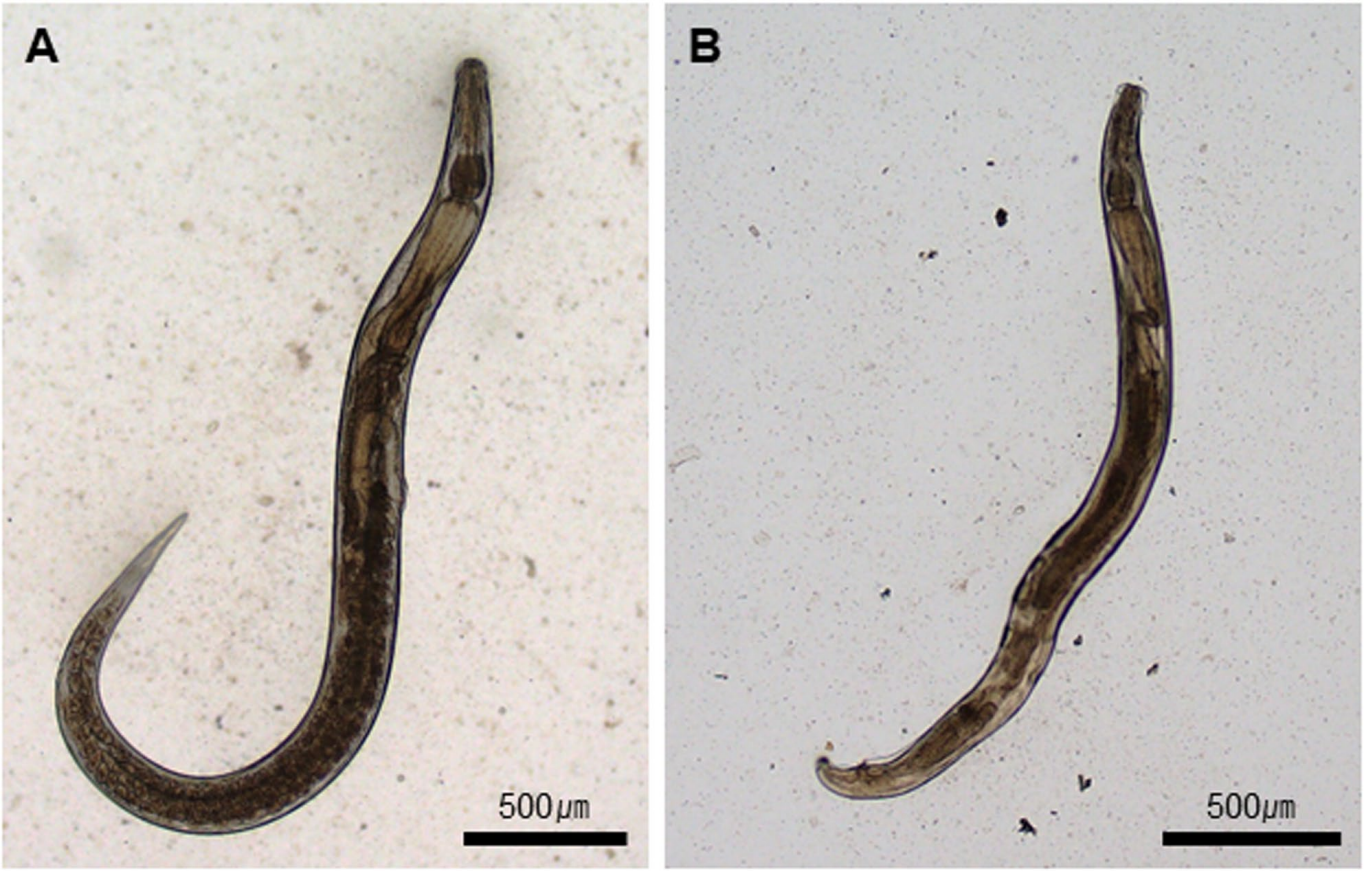
Microscopic examination of adult *Aspiculuris tetraptera* worms. (**A**) Female adult *A. tetraptera* containing eggs. (**B**) Male adult *A. tetraptera*. Scale bars = 500㎛

The pinworm outbreak in our facility was identified incidentally during an experiment. *A. tetraptera* infestation was monitored every two weeks for 12 weeks based on the pinworm life cycle, and subsequent monitoring continued for up to one year. The presence of *A. tetraptera* was confirmed through fecal flotation and post-mortem examination of adult worms in the large intestine (Fig. 1). For fecal flotation, fresh stool samples were collected from each mouse in every cage. The stool was diluted in a saturated NaCl solution (6.14 M) and filtered through medical gauze to remove debris. The filtered solution was transferred into a 15-mL conical tube, and additional NaCl solution was added until the liquid slightly exceeded the rim. A coverslip was placed on the top of the tube and left for 10 min. The coverslip was then transferred onto a slide glass for microscopic examination of eggs under a light microscope (Fig. 1A). For adult worm identification, necropsy was performed. Following euthanasia, the large intestines of the sacrificed mice were excised, cut longitudinally, and agitated in phosphate-buffered saline (PBS). The intestinal contents were examined for the presence of adult worms (Fig. 1B), which were also observed under the microscope (Fig. 2).

### Ivermectin treatment

Treatment for *A. tetraptera* infestation involved both injectable and spray applications of ivermectin (Eagle Vet, Korea). Each mouse received a subcutaneous injection of ivermectin at a dose of 1 mg/kg, diluted in 0.9% normal saline. Injections were administered every two weeks for a total of three doses, concluding at week 4. In parallel, ivermectin spray was applied directed to each mouse following environmental decontamination procedures. For environmental decontamination, ivermectin was sprayed at a concentration of 8.4 mg/L onto housing cages containing mice, as well as racks and ventilators, once weekly. The facility was additionally disinfected using a 1:300 dilution of commercial bleach, and bedding was replaced weekly. Regular diet and water provision remained unchanged. The decontamination process was conducted weekly for four consecutive weeks.

## Results

After the initial diagnosis of *A. tetraptera* at week 0, ivermectin treatment was initiated. The drug was administered every two weeks for a total of three doses, with the final treatment at week 4. The treatment timeline and outcomes are summarized in Table 2. Before treatment, all immunodeficient mouse groups tested positive for both eggs and adult worms. By week 2, all cages tested negative for both, and this status remained consistent throughout the treatment period. Following the cessation of treatment at week 4, all diagnostic tests continued to yield negative results for both eggs and adult worms over the subsequent 50 weeks. Pinworm monitoring was discontinued after one year. No mortality was observed during the ivermectin treatment.

**Table 2:** Treatment outcomes over time in different immunodeficient mouse models

Weeks Post-Treatment Mouse Strain	0	2	4	6	8	10	12	24	50
Rag1^−/−^ mouse	+	-	-	-	-	-	-	-	-
Rag2^−/−^ mouse	+	-	-	-	-	-	-	-	-
C1qa^−/−^ mouse	+	-	-	-	-	-	-	-	-
Rag1^−/−^, C1qa^−/−^ mouse	+	-	-	-	-	-	-	-	-
Rag2^−/−^, C1qa^−/−^ mouse	+	-	-	-	-	-	-	-	-
TLR4^−/−^, TLR9^−/−^ mouse	+	-	-	-	-	-	-	-	-
TNF-α^−/−^ mouse	+	-	-	-	-	-	-	-	-
IL-10^−/−^ mouse	+	-	-	-	-	-	-	-	-
IL2RG^−/−^ mouse	+	-	-	-	-	-	-	-	-

+, positive for eggs and adults; -, negative for eggs and adults

## Discussion

Pinworm infections remain a persistent issue in laboratory mouse colonies, even within SPF facilities, due to the environmental stability and transmissibility of pinworm eggs [11, 12]. Although pinworm infections generally have minimal effects on host health, they are often considered less significant than major pathogens such as viruses and bacteria, leading to delayed detection. Additionally, eradication can be challenging due to the complex life cycle of pinworms, highlighting the need for a rapid and efficient elimination strategy [13].

Ivermectin is one of the most widely used anthelmintics for treating pinworm infestations in laboratory animal facilities, owing to its affordability and accessibility [14]. Various ivermectin-based treatment regimens in laboratory rodents are summarized in Table 3. The most common approach is to deliver ivermectin in drinking water for at least three weeks [14, 16, 17]. Some protocols also incorporate piperazine in combination with ivermectin [18, 19]. However, several studies report that ivermectin alone is sufficient to eliminate pinworm infections in laboratory rodents. Effective eradication generally requires a minimum of three weeks of treatment, corresponding to the life cycle of the pinworm; the prepatent period of *A. tetraptera* is approximately three weeks [20]. In addition to pharmacological intervention, strict environmental control is essential for the successful management and prevention of reinfection.

**Table 3:** Ivermectin-based treatment strategies for pinworm infestations in laboratory rodents

Reference	Rodents	Targeted pinworms	Treatment regimen	Treatment duration	Environmental control
This paper	Mice	*A. tetraptera*	1 mg/kg ivermectin subcutaneous injection, every two weeks; 8.4 mg/L ivermectin spray, once a week	4 weeks	Bedding and cages changed weekly, facility and equipment sprayed with 1:300 bleach solution once weekly
Sueta, 2002 [15]	Mice	*S. obvelata*,* A. tetraptera*	1% ivermectin spray, once a week	3 weeks	Bedding and cages changed weekly
Klement, 1996 [16]	Mice, rats	*S. obvelata*,* S. muris*	Ivermectin in drinking water, 4 days/week; 25 mg/L (rats), 8 mg/L (mice)	4 weeks	Cages changed twice weekly
Hickman, 2008 [14]	Mice	*A. tetraptera*	Ivermectin in drinking water, 4 days/week; 8.4 mg/L	7 weeks	Facility cleaned with 10% bleach once weekly
Lytvynets, 2010 [17]	Rats	*S. muris*	Ivermectin in drinking water, every fifth day; 2.5 mg/kg	20 days	Bedding and cages changed every fifth day
Zenner, 1998 [18]	Mice, rats	*S. muris*,* A. tetraptera*	Piperazine in drinking water (2.1 mg/ml) on week 0–1; ivermectin in drinking water (0.007 mg/ml) on week 2–3; piperazine in drinking water (2.1 mg/ml) on week 4–5	6 weeks	Cages cleaned with detergent, equipment autoclaved
Krishnaveni, 2019 [19]	Mice, rats	*Syphacia* spp., *A. tetraptera*	Piperazine in drinking water (10.8 mg/ml) on week 0–1; ivermectin in drinking water (0.01 mg/ml) on week 2–3	4 weeks	Not specified

In this study, we employed subcutaneous injection as the method of ivermectin administration for treating pinworm infections. This approach ensures precise dosing and rapid drug distribution [21]. Also, the absence of mortality during the treatment period in our study supports the relative safety of ivermectin use in various knockout mouse models, although other immunological or physiological parameters were not included. However, subcutaneous administration requires specialized techniques and can be labor-intensive, particularly when applied to large mouse colonies. As such, this method is more recommendable for smaller colonies. Nonetheless, it is important to note that research on the effects of ivermectin administration in pregnant or lactating mice remains limited, necessitating caution in these populations [22].

Before achieving successful eradication of pinworms, we initially attempted a regimen involving subcutaneous ivermectin injections every three weeks in combination with weekly spray application over five weeks (data not shown). However, pinworm reinfection occurred several weeks later, indicating that neither subcutaneous injection at three-week intervals nor weekly spray alone was sufficient for complete elimination of pinworms in mice. This outcome contrasts with the findings of Sueta et al. [15], whose study demonstrated efficacy with weekly spray for only three weeks. This discrepancy may be attributed to differences in experimental conditions; specifically, Sueta et al. followed a strictly controlled experimental schedule, whereas our treatment was applied under practical, non-experimental conditions. Moreover, spray application does not allow for accurate dosing, which may further reduce its reliability. Our findings suggest that subcutaneous ivermectin administration at two-week intervals is optimal for effective treatment. In addition, ivermectin spray alone should not be relied upon for complete eradication of pinworms within a short time frame.

In conclusion, we report the successful eradication of *A. tetraptera* in multiple knockout mouse models through a four-week treatment regimen combining subcutaneous ivermectin administration with spray application. Long-term use of ivermectin has been associated with behavioral side effects in rodents, including alterations in locomotion and neuropsychiatric behaviors [23, 24]. Notably, knockout mice deficient in *mdr1a/b* P-glycoprotein and *bcrp* exhibit increased concentrations of ivermectin in the brain due to compromised blood-brain barrier function [25]. These findings underscore the importance of considering both the duration of ivermectin exposure and the genetic background of the animals when designing treatment protocols. Given the potential risks of prolonged ivermectin use in laboratory colonies, achieving complete eradication within a short timeframe is particularly significant. However, this report is based on a single case with a small sample size and lacks a comprehensive evaluation of physiological and metabolic safety in immunodeficient mice. Therefore, the conclusions should be interpreted with caution, and broader validation will be required. Furthermore, ongoing facility management and routine health monitoring are essential for long-term prevention and the maintenance of environmental stability.

## Conclusions

Although ivermectin-based pinworm eradication has been well-documented in general mouse colonies, its efficacy in immunodeficient models remains largely unexplored. This case study demonstrates the successful elimination of *A. tetraptera* in various immunodeficient mice using ivermectin, with no observed mortality. However, further research is warranted to assess the physiological and metabolic effects of ivermectin in specific immunodeficient models.

## Data Availability

The data used in this current study are available from the corresponding author upon reasonable request.

## Abbreviations

*A. tetraptera*: *Aspiculuris tetraptera*
SPF: Specific pathogen-free

## References

[1] Baker DG. Natural pathogens of laboratory mice, rats, and rabbits and their effects on research. Clin Microbiol Rev. 1998;11(2):231–66. 9564563 10.1128/cmr.11.2.231 PMC106832

[2] Nicklas W, Baneux P, Boot R, Decelle T, Deeny AA, Fumanelli M, et al. Recommendations for the health monitoring of rodent and rabbit colonies in breeding and experimental units. Lab Anim. 2002;36(1):20–42. 11831737 10.1258/0023677021911740

[3] Burkholder T, Foltz C, Karlsson E, Linton CG, Smith JM. Health evaluation of experimental laboratory mice. Curr Protoc Mouse Biol. 2012;2:145–65. 22822473 10.1002/9780470942390.mo110217 PMC3399545

[4] Chen XM, Li X, Lin RQ, Deng JY, Fan WY, Yuan ZG, et al. Pinworm infection in laboratory mice in Southern China. Lab Anim. 2011;45(1):58–60. 21138918 10.1258/la.2010.009135

[5] Pritchett-Corning KR, Cosentino J, Clifford CB. Contemporary prevalence of infectious agents in laboratory mice and rats. Lab Anim. 2009;43(2):165–73. 19015179 10.1258/la.2008.008009

[6] Albers TM, Henderson KS, Mulder GB, Shek WR. Pathogen prevalence estimates and diagnostic methodology trends in laboratory mice and rats from 2003 to 2020. J Am Assoc Lab Anim Sci. 2023;62(3):229–42. 37127407 10.30802/AALAS-JAALAS-22-000097 PMC10230541

[7] Whary MT, Baumgarth N, Fox JG, Barthold SW. Biology and diseases of mice. In: Fox JG, Anderson LC, Otto G, Pritchett-Corning KR, Whary MT, editors. Laboratory animal medicine. 3rd ed. Amsterdam: Academic; 2015. pp. 43–149.

[8] Wendt S, Trawinski H, Schubert S, Rodloff AC, Mössner J, Lübbert C. The diagnosis and treatment of pinworm infection. Dtsch Arztebl Int. 2019;116(13):213–19. 31064642 10.3238/arztebl.2019.0213 PMC6522669

[9] Fitch BA, Situ J, Wiemels JL, Kogan SC, Zhou M. Impact of pinworm infection on the development of murine B-cell leukemia/lymphoma in the presence and absence of *ETV6::RUNX1*. Haematologica. 2023;108(12):3480–4. 37345483 10.3324/haematol.2022.282591 PMC10690896

[10] Michels C, Goyal P, Nieuwenhuizen N, Brombacher F. Infection with *Syphacia obvelata* (pinworm) induces protective Th2 immune responses and influences ovalbumin-induced allergic reactions. Infect Immun. 2006;74(10):5926–32. 16988272 10.1128/IAI.00207-06 PMC1594938

[11] Jacoby RO, Lindsey JR. Risks of infection among laboratory rats and mice at major biomedical research institutions. ILAR J. 1998;39(4):266–71. 11528087 10.1093/ilar.39.4.266 PMC7537657

[12] Otto GM, Franklin CL, Clifford CB. Biology and diseases of rats. In: Fox JG, Anderson LC, Otto G, Pritchett-Corning KR, Whary MT, editors. Laboratory animal medicine. 3rd ed. Amsterdam: Academic; 2015. pp. 151–207.

[13] Baker DG. Parasites of rats and mice. In: Baker DG, editor. Flynn’s parasites of laboratory animals. 2nd ed. Ames, Iowa: Blackwell; 2007. pp. 303–97.

[14] Hickman D, Swan M, Hartman GP. A cost-effective and efficacious method of pinworm treatment for large colonies of mice. Lab Anim (NY). 2008;37(7):308–12. 18568009 10.1038/laban0708-308 PMC7091669

[15] Sueta T, Miyoshi I, Okamura T, Kasai N. Experimental eradication of pinworms (*Syphacia obvelata* and *Aspiculuris tetraptera*) from mice colonies using Ivermectin. Exp Anim. 2002;51(4):367–73. 12221930 10.1538/expanim.51.367

[16] Klement P, Augustine JM, Delaney KH, Klement G, Weitz JI. An oral Ivermectin regimen that eradicates pinworms (*Syphacia* spp.) in laboratory rats and mice. Lab Anim Sci. 1996;46(3):286–90. 8799934

[17] Lytvynets A, Langrová I, Lachout J, Vadlejch J, Fučíková A, Jankovská I. Drinking water Ivermectin treatment for eradication of pinworm infections from laboratory rat colonies. Helminthologia. 2010;47:233–37.

[18] Zenner L. Effective eradication of pinworms (*Syphacia muris, syphacia obvelata* and *Aspiculuris tetraptera*) from a rodent breeding colony by oral anthelmintic therapy. Lab Anim. 1998;32(3):337–42. 9718483 10.1258/002367798780559202

[19] Krishnaveni N, Sandhya S, Rosa JS, Shrruthi BM, Ramachandra SG. Effective treatment regimen for control and eradication of oxyurids in laboratory rodents. J Lab Anim Sci. 2019;2(1):38–41.

[20] Taffs LF. Pinworm infections in laboratory rodents: a review. Lab Anim. 1976;10(1):1–13. 768631 10.1258/002367776780948862

[21] Sharun K, Shyamkumar TS, Aneesha VA, Dhama K, Pawde AM, Pal A. Current therapeutic applications and Pharmacokinetic modulations of Ivermectin. Vet World. 2019;12(8):1204–11. 31641298 10.14202/vetworld.2019.1204-1211 PMC6755388

[22] Westlake CS, Aronoff DM. Evaluating the risks of systemic maternal Ivermectin exposure during pregnancy in human and vertebrate animals: A scoping review. Curr Drug Saf. 2021;16(3):299–308. 33109066 10.2174/1574886315999200820125001

[23] Davis JA, Paylor R, McDonald MP, Libbey M, Ligler A, Bryant K, et al. Behavioral effects of Ivermectin in mice. Lab Anim Sci. 1999;49(3):288–96. 10403444

[24] Comis-Neto AA, Jardim NS, Quines CB, Bianchini MC, Gomes J, Batista WT, et al. Repeated oral administration of Ivermectin belatedly induces toxicity and disrupts the locomotion and neuropsychiatric behavior in rats. ACS Omega. 2025;10(13):12993–3001. 40224401 10.1021/acsomega.4c09536 PMC11983209

[25] Geyer J, Gavrilova O, Petzinger E. Brain penetration of Ivermectin and selamectin in mdr1a,b P-glycoprotein- and bcrp- deficient knockout mice. J Vet Pharmacol Ther. 2009;32(1):87–96. 19161460 10.1111/j.1365-2885.2008.01007.x

